# Some Remarks on the Operation for Hernia*Read before the Austin District Medical Society.

**Published:** 1905-01

**Authors:** T. J. Bennett

**Affiliations:** Austin, Texas


### TEXAS MEDICAL JOURNAL.

ESTABLISHED JULY, 1885.

PUBLISHED MONTHLY.—SUBSCRIPTION $1.00 A YEAR.

VOL. XX. AUSTIN, JANUARY, 1905. No. 7.

### Original Contributions.

For Texas Medical Journal.

### Some Remarks on the Operation for Hernia.*

BY T. J. BENNETT, M. D., AUSTIN, TEXAS.

Herniotomy for some reason has never been a popular operation in this section of the country,—in fact, it has hardly been kept up to the times. In some portions of the North and East this operation has run into almost a fad, like the operation for appendicitis, and many of the most eminent authorities are as radical in advising operation for the one as the other—"in every case as soon as the diagnosis is made."

The perfection to which the Bassini operation and its modifications has been brought would seem to justify such advice. During the last few years, by this method mainly, the mortality has been brought down to less than 1 per cent, one-half to one-ninth, and the relapses to less than 5 per cent. These statistics, brought out chiefly by Bull and Coley, refer to simple inguinal hernia, and not to strangulated hernia. Such statistics come from the hands of clean and careful operators, but with the ordinary regard for asepsis and a clear interest in the detail, as good success follows the operations of the country physician as the surgeon in the larger cities. Herniotomy, therefore, is as safe an operation as any in the catalog of surgery.

The impression that this is a dangerous and difficult operation to

*Read before the Austin District Medical Society.

perform has caused many a good man to go through life troubled with a truss, and subjected to greater danger from strangulation than the simple Bassini in the hands of a clean surgeon with a little experience.

The young or the old are scarcely barred from the benefits of the operation. In the very young, however, many cures result from the employment of properly fitting trusses, and the very old hardly see proper to undergo any kind of an operation, unless they are bed-ridden from an unrestrained hernia. A very good illustration in point is an operation performed two years ago on an old man, age 82 at the time, an inmate of the Confederate Home, who had been confined to his bed for a long time from an unrestrained inguinal hernia of the left side. The opening in the flank measured two by six or seven inches, which precluded any possible benefits from a truss. The abdominal walls were remarkably free from muscular tissue and fascia—were a soft, flabby stratum of fat and skin. Still the results were good, and the old man, in two or three weeks, was up and about, and has remained so ever since.

The essential features of the operation, or, rather, the points to be regarded most sincerely, can be summarized as follows:

1. The preparation of the patient.
2. The securing of perfect asepsis and primary union.
3. Careful coaptation and loose suturing.
4. Recumbent position and good support of wound by adhesive plaster.
5. Short anesthesia.

First. The patient should be relieved of any cough that may be troubling him, as this is one of the great sources of intra-abdominal pressure, and unless guarded against by previous treatment, a failure may result from this cause alone. A good purgative should be given on the night before the operation. The pubis and field of operation should be shaven and thoroughly scrubbed with green soap and antiseptics, and a moist bichloride gauze dressing should be applied for at least twenty-four hours before the operation. If plenty of time is at hand, such preliminary measures may be continued forty-eight hours instead of twenty-four.

Second. Without perfect asepsis it is not expected to obtain primary union, and without primary union it is not expected to obtain a strong "muscular contractile barrier against future relapses." Over-manipulation of delicate structures conduces to failure in the union; suppurations from septic ligatures or other causes conduce also to a failure in the union by producing cicatricial tissue formation. It is essential, therefore, that the means for securing these ends should have our first consideration.

Third. Perfect coaptation of stable parts is absolutely necessary to good results. Fat or loose connective tissue stitched to Poupart's ligament instead of stitching the internal oblique and conjoined tendon would result in failure in nearly every case. All such unstable tissue should be trimmed out, and the coaptation of none but strong muscle and fascia should be sutured. It is also essential, for the same reason, that blood clots should be carefully sponged out and all bleeding securely checked,—in fact, it is a point well worth considering, to prevent, as far as possible, the covering of the tissues with blood during the progress of the operation. Such blood stain not only obscures the field and makes undue manipulation necessary, but the minute coagula prevent the early firm union most desirable in herniotomy.

Another very common source of failure in this operation is tight suturing. The very nature of the structures to be united demands loose suturing. The parts should be drawn together just tight enough to prevent their sliding past each other. The oedema, which is always present, must be provided for by that means. The absorbable ligatures which are now universally employed in herniotomy yield to a certain extent, but this is not sufficient to prevent strangulation and cutting of the tissues.

To witness Ochsner perform this operation, as I have recently done, one almost shudders at the loose suturing. Opposing structures are made to barely touch. Still there are his results. None better in any country.

Fourth. After the operation has been finished, it is essential to support the wound with strong adhesive strips to provide against intra-abdominal pressure caused by vomiting, coughing, etc. The patient should be kept in the recumbent position for two or three weeks, and the bowels should be kept open.

Fifth. The stage of anesthesia should be as short as possible on account of vomiting, which is apt to follow the prolonged administration of chloroform or ether. This advice is valuable anywhere in surgery, but it is especially applicable in herniotomy.

Another good point in surgery is always to have the anatomy of the operation well in hand. But there are instances in the Bassini where one must do the operation and consider the anatomy afterwards. In very old or very emaciated subjects all muscular tissue may have taken its flight. The aponurotic fascia is only recognized, in such patients, by its being a trifle stronger than the connective tissue and fat. Time, therefore, should not be wasted in trying to determine the proper name of all the structures concerned in every operation. It seems to be quite sufficient to transplant the cord by attaching the most reliable tissues to Poupart's ligament and then covering the cord with what is left. Where it can be determined, however, the external oblique muscle should cover the cord, but if this muscle is not recognizable, what passes for it should go under the cord, because the weaker opening in such cases demand the greater reinforcement, the connective tissue and skin being sufficient with which to cover the cord. Then if all the other steps of the operation are faultless, success is assured.

When the patient is ready to be on his feet the question of a truss will arise—shall one be worn or not? The practice of those with greatest experience in this operation is widely divergent. Bull and Coley never advise a truss in children after operation, and the time confined in the recumbent position is only two to two and a half weeks. In adults, these gentlemen seldom advise the use of a truss. When the patient is beyond middle age, the opening large, and the abdominal walls flabby and scant, in muscle and fascia, a soft truss may be worn. I think it good advice to begin the use of a truss as soon as the signs of recurrence appear, since a new sac or canal has to be made by a slow protrusion. This is Lockwood's advice, and it occurs to me as conservative and practical.

In reference to strangulated hernia, the same general course should be adopted in the operation, except one is dealing with a constricted inflammatory condition of the protruding parts. Just how to manage a case of strangulated hernia, calls forth the best judgment. In relieving the constriction one must determine, if possible, what kind of a hernia he is considering; if indirect, he must cut outward; if direct, he must cut inward, so as to avoid the deep epigastric artery. If he can not make out a clear case, he had better nick upwards and be careful. If the protruding parts are gangrenous, they should be trimmed off, after relieving the constriction, and stitched temporarily to the wall and results awaited. If it is a question whether the parts are dead, the constriction should be relieved and the parts left outside long enough to determine their condition. The length of time that a constricted hernia may exist without producing gangrene depends largely upon the vitality of the organism to start with and upon the degree of constriction. Operations for strangulated hernia should be performed as soon as possible after reduction has failed.

